# Intracranial Germinoma Misdiagnosed as Hyperthyroidism: A Case Report and Review of the Literature

**DOI:** 10.3389/fendo.2021.789109

**Published:** 2022-01-24

**Authors:** Juan Tian, Jialu Wu, Zhe Yan, Hui Huang

**Affiliations:** Department of Endocrinology and Metabolism, West China Hospital, Sichuan University, Chengdu, China

**Keywords:** intracranial germinoma, hyperthyroidism, hypopituitarism, central diabetes insipidus, radiotherapy

## Abstract

Intracranial germ cell tumors (GCTs) are relatively rare, which account for 0.5% of all primary intracranial neoplasms. Intracranial germinomas most commonly occur in the pineal and suprasellar region, making up the majority of all intracranial GCTs. For its diversified clinical manifestations, the diagnosis is easily confused with other diseases. Here, we present a case of a 19-year-old boy with intracranial germinoma who was preliminarily misdiagnosed as hyperthyroidism for the symptoms of weight loss and thyroid dysfunction.

## Background

Primary intracranial germ cell tumors (GCTs) originate from primordial germ cells. Germinomas comprise the majority of GCTs and usually develop in the midline structures, especially in the pineal followed by the suprasellar region (1). They mainly affect children and young adults, and have a male predominance (2). Depending on the size and location of the GCTs, there are different clinical manifestations: hypopituitarism, diabetes insipidus, intracranial hypertension, etc. The diagnosis of intracranial germinoma is easily confused with other diseases due to its diverse clinical manifestations. Here, we report a case of intracranial germinoma in a 19-year-old boy who was misdiagnosed as hyperthyroidism.

## Case Presentation

A 19-year-old boy was admitted to the hospital for complaints of fatigue, poor appetite, and weight loss without headache, nausea, vomiting, polydipsia, and polyuria. Pre-admission thyroid hormones determination: thyroid stimulating hormone(TSH) <0.005 mU/L (Reference range 0.27–4.2 mU/L), free triiodothyronine (FT3) 7.66 pmol/L (Reference range 3.60–7.50 pmol/L), free thyroxine (FT4) 19.52 pmol/L (Reference range 12.0–22.0pmol/L). The preliminary diagnosis was hyperthyroidism.

Physical examination: T: 36.5°C, R: 18 bpm, BP: 85/52 mmHg, HR: 87 bpm, Height: 173 cm, Weight: 44 kg, BMI: 14.7kg/m^2^. Clear consciousness, dry skin, and normal development. Neurological examination was negative.

**Figure 1:**
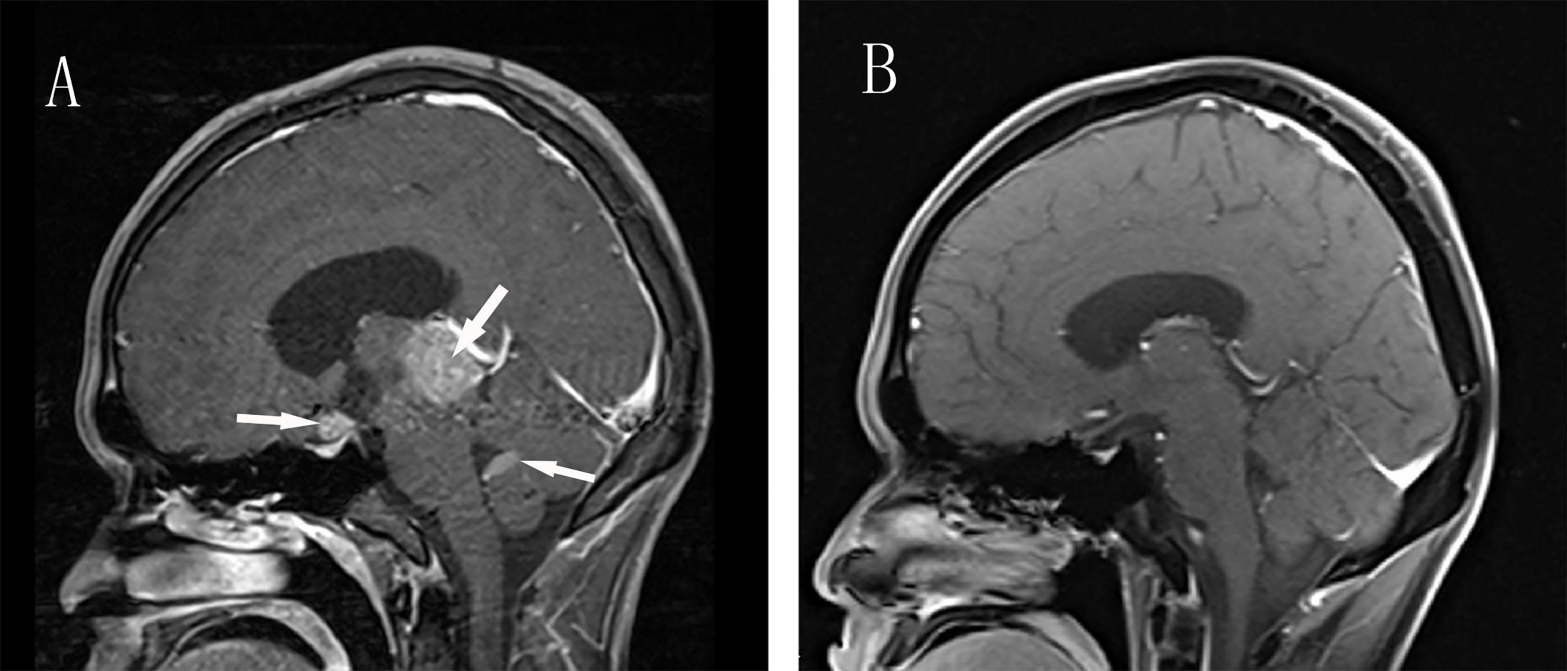
Enhanced Brain MRI before and after Radiotherapy. **(A)** Sagittal contrast-enhanced T1-weighted MR image revealing three masses in the pineal, suprasellar region and fourth ventricle(arrows). **(B)** After radiotherapy, T1-weighted MR image show total disappearance of the lesions.

Laboratory examination: blood glucose, hepatic function, renal function, routine blood count, and stool routine were normal. Redetermination of thyroid hormone on admission: TSH <0.005 mU/L, FT3 6.93 pmol/L, FT4 18.03 pmol/L. TSH receptor antibody (TRAb), thyroglobulin antibody (TGAb), and thyroid peroxidase antibody (TPOAb) were negative. The thyroid function of the patient was changing without any drug intervention over time (**Table 1**). Single-photon emission computed tomography (SPECT) thyroid imaging revealed decreased thyroid uptake of technetium. The results of additional hormone test showed secondary hypoadrenocorticism, secondary hypogonadism, and hyperprolactinemia (**Table 2**). Then, the patient was treated with hydrocortisone 50mg per day. A few days later, the patient began to complain about polydipsia and polyuria. The serum sodium concentration increased from 142 to 158 mmol/L and there was no change in urine specific gravity (1.004) during the water deprivation test, while a great increase in urine specific gravity (increased from 1.004 to 1.018) was observed after administration of desmopressin, this confirmed central diabetes insipidus (CDI). His polydipsia and polyuria were relieved by desmopressin. Contrast-enhanced MRI revealed nodular signals in the pineal, suprasellar region, and fourth ventricle (1.0, 2.7, and 1.1 cm in diameter, respectively) (**Figure 1A**). A spine MRI excluded metastatic lesions. Serum β-human chorionic gonadotropin (β-HCG) was 8.56 IU/L (Reference range <3.81 IU/L)and serum Alpha-fetoprotein (α-FP) was within the normal reference range. Consequently, the diagnosis of intracranial germinoma was considered. After the completion of diagnostic radiation therapy of 20 Gy and subsequent radiotherapy(the patient received three-dimensional conformal radiotherapy and the total dose was 40 Gy in fractions of 1.8–2.0 Gy per day, 5 d/wk), the lesions of the suprasellar, the pineal, and fourth ventricle almost disappeared (**Figure 1B**). Redetermination of the thyroid axis revealed that all the indices gradually returned to the normal range, with ACTH fluctuating around the lower limit of the reference range and low levels of cortisol. The patient was treated with hydrocortisone 20 mg/day and desmopressin 0.05 mg per day for cortisol replacement and CDI, respectively. His symptoms improved significantly, the 24-hour intake and output were maintained at about 2,000 ml, blood pressure returned to normal, and his weight increased. No recurrence occurred after a follow-up period of one year.

**Table 1:** Serum thyroid hormones with reference range.

Serum Thyroid Hormones	TSH	T3	FT3	T4	FT4
Reference range	0.27–4.2mU/L	1.3–3.1nmol/L	3.60–7.50pmol/L	62–164nmol/L	12.0–22.0pmol/L
On admission	<0.005		6.93		18.03
1 week later	0.006		3.84		14.88
2 weeks later	0.012		3.21		14.19
3 weeks later	0.018	1.19	3.66	86.22	12.48
2 weeks after RT	0.058	0.86	2.72	73.63	10.67
1 month after RT	0.765		2.76		11.33
2 months after RT	0.883	1.20	3.22	88.63	12.64
4 months after RT	1.630		3.59		14.67

TSH, thyroid stimulating hormone; T3, triiodothyronine; FT3, free triiodothyronine; T4, thyroxine; FT4, free thyroxine; RT, radiotherapy.

**Table 2:** Hormone test results.

Hormones	Lab value	Reference range
ACTH (8:00 A.M.)	10.87	5.00–78.00 ng/L
Cortisol (8:00 A.M.)	50.79	147.30–609.30 nmol/L
GH	2.10	0.03–2.47 ng/ml
LH	<0.10	1.70–8.60 mIU/L
FSH	<0.10	1.50–12.40 mIU/L
Estradiol	<5.00	25.80–60.70 pg/ml
Testosterone	1.99	0.28–11.10 ng/ml
PRL	78.22	4.60–21.40 ng/mL

ACTH, adrenocorticotropic hormone; GH, growth hormone; LH, luteinizing hormone; FSH, follicle-stimulating hormone; PRL, prolactin.

## Discussion

Primary central nervous system (CNS) germ cell tumors (GCTs) are rare, which represent approximately 0.5% of all primary intracranial neoplasms (3). These rare tumors primarily affect children (especially 10–14 years old) and young adults with a male preponderance (2, 3). The majority of intracranial GCTs appear in the midline structures of the brain, such as the pineal and suprasellar region (1). CNS GCTs include a heterogeneous group of neoplasms, which are commonly classified into germinomas and non-germinomatous germ cell tumors (NGGCTs) (4, 5). Germinomas comprise the largest proportion of CNS GCTs and show high radiosensitivity and also excellent prognosis. The optimal treatment is either radiotherapy alone or chemotherapy followed by radiotherapy (3, 6).

The diversity of clinical manifestations is related to the size and location of tumors. Tumors occurring in the pineal region can easily block the midbrain aqueduct, causing obstructive hydrocephalus, high intracranial pressure, Parinaud’s syndrome, etc. (7), while suprasellar tumors mostly lead to hypothalamo-hypophyseal insufficiency with corresponding clinical manifestations, namely, delayed growth, delayed or precocious puberty, central diabetes insipidus, fatigue, weight loss, etc. (8). This patient showed symptoms of fatigue and weight loss, had low levels of TSH and normal levels of FT4 and FT3, so subclinical hyperthyroidism was considered. However, we noticed the patient paradoxically had poor appetite and low blood pressure, instead of hypermetabolic symptoms such as heat intolerance, sweating, and increased appetite. Additionally, during serial follow-up of the thyroid hormone after admission, the FT3 and FT4 levels were on a downward trend. The results of the pituitary hormone test showed he had secondary hypoadrenocorticism, secondary hypogonadism, and hyperprolactinemia. In the absence of cortisol, the symptoms of polydipsia and polyuria of the patients with diabetes insipidus cannot be obvious. After being given hydrocortisone, the symptoms of polydipsia and polyuria become apparent for an increase in blood volume (9). The subsequent water deprivation vasopressin test confirmed central diabetes insipidus. The patient had normal physical development, which might suggest the tumor had been present and developed after his puberty.

Although histopathology is the golden standard for the diagnoses of CNS GCTs, it is difficult to obtain the pathological specimen clinically. Imaging examination is helpful in diagnosis, typical MRI abnormalities in typical locations are strongly suggestive of intracranial GCTs. Intracranial GCTs lesions that involve both the pineal gland and suprasellar region are frequently termed as bifocal GCTs (10), of which the majority are germinomas (11). Synchronous neoplasms in any other location such as the fourth ventricle are considered to represent disseminated foci (12). MRI examinations of the patient revealed localized lesions were distributed in the pineal, suprasellar region, and fourth ventricle and presented significantly heterogeneous enhancement. Alpha-fetoprotein (α-FP) and Beta-Human chorionic gonadotropin (β-hCG) are two markers of GCTs, which are not produced by any other primary intracranial neoplasms (13). β-hCG is more valuable in diagnosing germinomas, because germinomas may secrete low levels of β-hCG instead of α-FP (14). Hu et al. suggest β-hCG ≥8.2 IU/L in CSF or serum β-hCG ≥2.5 IU/L as cutoff values for the clinical diagnosis of intracranial GCTs (15). In our patient, the high serum β-hCG (8.56 IU/L) and normal α-FP further support the diagnosis.

Because of the high radiosensitivity of germinomas compared with other intracranial tumors, diagnostic radiotherapy with a dose of 20 Gy was once used without histological verification (16–18). Prompt response to low-dose radiation (tumor can be reduced in mean diameter by more than 80% at 15-20 Gy) was deemed as one of the criteria for the clinical diagnosis of intracranial germinoma (19, 20).

For CNS germinomas, there has been consensus that radiotherapy should be the first line treatment and adjuvant chemotherapy could be conducive to the reduction of radiation dosage, while surgical resection plays a limited role (21, 22). The favored management for patients with symptomatic obstructive hydrocephalus is endoscopic third ventriculostomy (ETV) (22). Craniospinal irradiation (CSI) with focal boosts to tumor sites remains the standard of care for metastatic germinoma. The SIOP CNS GCT 96 study demonstrated 98% overall survival at 5 years with a CSI dose of 24 Gy followed by a 16 Gy boost to the tumor sites, no case of relapse was reported during a median follow-up of 6 years, and there was no additional benefit of chemotherapy (23). A retrospective review presented 10 patients with histologically proven primary intracranial germinoma who were treated by low-dose CSI with local boosts to a total dose of 40 Gy, all patients were alive with a median follow up time of 10.9 years, none with relapsed disease (24).

After trial therapy with a dose of 20 Gy and subsequent radiation, significant shrinkage of the lesions of the patient was observed. As the tumor volume shrunk obviously, his thyroid function gradually returned to normal. It suggested that the changes in his thyroid function were related to transient hyposecretion of TSH caused by tumor compression, which was misdiagnosed as subclinical hyperthyroidism.

## Conclusion

Because germinomas occur at different ages with different course and lesion sites, the clinical manifestations and lab results are diverse and deceptive. This patient was misdiagnosed as subclinical hyperthyroidism for TSH reduction at first. Therefore, it is of great help to the diagnosis by dynamic observation of the changes in symptoms and lab results. By the way, imaging examination, β-hCG test and diagnostic radiotherapy are valuable for the diagnosis of germinoma.

## Data Availability Statement

The original contributions presented in the study are included in the article/supplementary material. Further inquiries can be directed to the corresponding author.

## Ethics Statement

Written informed consent was obtained from the individual(s) for the publication of any potentially identifiable images or data included in this article.

## Author Contributions

JT wrote the manuscript. JW and ZY were responsible for the collection of the clinical data and the follow-up of the patient. HH designed and revised the manuscript. All authors contributed to the article and approved the submitted version.

## Funding

This work was supported by the Science & Technology Department of Sichuan Province, China (Grant No. 2020YFS0193).

## Conflict of Interest

The authors declare that the research was conducted in the absence of any commercial or financial relationships that could be construed as a potential conflict of interest.

## Publisher’s Note

All claims expressed in this article are solely those of the authors and do not necessarily represent those of their affiliated organizations, or those of the publisher, the editors and the reviewers. Any product that may be evaluated in this article, or claim that may be made by its manufacturer, is not guaranteed or endorsed by the publisher.

## References

[1] Matsutani M Sano K Takakura K Fujimaki T Nakamura O Funata N. Primary Intracranial Germ Cell Tumors: A Clinical Analysis of 153 Histologically Verified Cases. J Neurosurg (1997) 86(3):446–55. doi: 10.3171/jns.1997.86.3.0446 9046301

[2] Gittleman H Cioffi G Vecchione-Koval T Ostrom QT Kruchko C Osorio DS. Descriptive Epidemiology of Germ Cell Tumors of the Central Nervous System Diagnosed in the United States From 2006 to 2015. J Neurooncol (2019) 143(2):251–60. doi: 10.1007/s11060-019-03173-4 31025275

[3] Thakkar JP Chew L Villano JL. Primary CNS Germ Cell Tumors: Current Epidemiology and Update on Treatment. Med Oncol (2013) 30(2):496. doi: 10.1007/s12032-013-0496-9 23436013

[4] Fujimaki T. Central Nervous System Germ Cell Tumors: Classification, Clinical Features, and Treatment With a Historical Overview. J Child Neurol (2009) 24(11):1439–45. doi: 10.1177/0883073809342127 19841431

[5] Louis DN Perry A Reifenberger G von Deimling A Figarella-Branger D Cavenee WK. The 2016 World Health Organization Classification of Tumors of the Central Nervous System: A Summary. Acta Neuropathol (2016) 131(6):803–20. doi: 10.1007/s00401-016-1545-1 27157931

[6] Bamberg M Kortmann RD Calaminus G Becker G Meisner C Harms D. Radiation Therapy for Intracranial Germinoma: Results of the German Cooperative Prospective Trials MAKEI 83/86/89. J Clin Oncol (1999) 17(8):2585–92. doi: 10.1200/JCO.1999.17.8.2585 10561326

[7] Dufour C Guerrini-Rousseau L Grill J. Central Nervous System Germ Cell Tumors: An Update. Curr Opin Oncol (2014) 26(6):622–26. doi: 10.1097/CCO.0000000000000140 25233069

[8] Arnaout MM Gerges MM Cummock MD El Asri AC Greenfield JP Anand VK. Endonasal Surgery for Suprasellar Germ Cell Tumors: Two Cases and Review of the Literature. Acta Neurochir (Wien) (2019) 161(8):1699–704. doi: 10.1007/s00701-019-03969-3 31214781

[9] Chin HX Quek TP Leow MK. Central Diabetes Insipidus Unmasked by Corticosteroid Therapy for Cerebral Metastases: Beware the Case With Pituitary Involvement and Hypopituitarism. J R Coll Physicians Edinb (2017) 47(3):247–9. doi: 10.4997/JRCPE.2017.307 29465100

[10] Lee L Saran F Hargrave D Bódi I Bassi S Hortobágyi T. Germinoma With Synchronous Lesions in the Pineal and Suprasellar Regions. Childs Nerv Syst (2006) 22(12):1513–8. doi: 10.1007/s00381-006-0248-7 17053934

[11] Takami H Fukuoka K Fukushima S Nakamura T Mukasa A Saito N. Integrated Clinical, Histopathological, and Molecular Data Analysis of 190 Central Nervous System Germ Cell Tumors From the iGCT Consortium. Neuro Oncol (2019) 21(12):1565–77. doi: 10.1093/neuonc/noz139 PMC6917411 31420671

[12] Weksberg DC Shibamoto Y Paulino AC. Bifocal Intracranial Germinoma: A Retrospective Analysis of Treatment Outcomes in 20 Patients and Review of the Literature. Int J Radiat Oncol Biol Phys (2012) 82(4):1341–51. doi: 10.1016/j.ijrobp.2011.04.033 21669501

[13] Bromberg JE Baumert BG de Vos F Gijtenbeek JM Kurt E Westermann AM. Primary Intracranial Germ-Cell Tumors in Adults: A Practical Review. J Neurooncol (2013) 113(2):175–83. doi: 10.1007/s11060-013-1114-6 23526409

[14] Allen J Chacko J Donahue B Dhall G Kretschmar C Jakacki R. Diagnostic Sensitivity of Serum and Lumbar CSF bHCG in Newly Diagnosed CNS Germinoma. Pediatr Blood Cancer (2012) 59(7):1180–2. doi: 10.1002/pbc.24097 PMC3356788 22302772

[15] Hu M Guan H Lau CC Terashima K Jin Z Cui L. An Update on the Clinical Diagnostic Value of β-hCG and αfp for Intracranial Germ Cell Tumors. Eur J Med Res (2016) 21:10. doi: 10.1186/s40001-016-0204-2 26968839 PMC4788851

[16] Shirato H Nishio M Sawamura Y Myohjin M Kitahara T Nishioka T. Analysis of Long-Term Treatment of Intracranial Germinoma. Int J Radiat Oncol Biol Phys (1997) 37(3):511–5. doi: 10.1016/s0360-3016(96)00607-4 9112446

[17] Linstadt D Wara WM Edwards MS Hudgins RJ Sheline GE. Radiotherapy of Primary Intracranial Germinomas: The Case Against Routine Craniospinal Irradiation. Int J Radiat Oncol Biol Phys (1988) 15(2):291–7. doi: 10.1016/s0360-3016(98)90007-4 3403312

[18] Dearnaley DP A'Hern RP Whittaker S Bloom HJ. Pineal and CNS Germ Cell Tumors: Royal Marsden Hospital Experience 1962-1987. Int J Radiat Oncol Biol Phys (1990) 18(4):773–81. doi: 10.1016/0360-3016(90)90396-2 2323968

[19] Shibamoto Y Sasai K Oya N Hiraoka M. Intracranial Germinoma: Radiation Therapy With Tumor Volume-Based Dose Selection. Radiology (2001) 218(2):452–6. doi: 10.1148/radiology.218.2.r01ja08452 11161161

[20] Ogawa K Shikama N Toita T Nakamura K Uno T Onishi H. Long-Term Results of Radiotherapy for Intracranial Germinoma: A Multi-Institutional Retrospective Review of 126 Patients. Int J Radiat Oncol Biol Phys (2004) 58(3):705–13. doi: 10.1016/j.ijrobp.2003.07.001 14967424

[21] Frappaz D Dhall G Murray MJ Goldman S Faure Conter C Allen J. Intracranial Germ Cell Tumors in Adolescents and Young Adults: European and North American Consensus Review, Current Management and Future Development. Neuro Oncol (2021) in press. doi: 10.1093/neuonc/noab252 PMC8972311 34724065

[22] Murray MJ Bartels U Nishikawa R Fangusaro J Matsutani M Nicholson JC. Consensus on the Management of Intracranial Germ-Cell Tumours. Lancet Oncol (2015) 16(9):e470–7. doi: 10.1016/S1470-2045(15)00244-2 26370356

[23] Calaminus G Kortmann R Worch J Nicholson JC Alapetite C Garrè ML. SIOP CNS GCT 96: Final Report of Outcome of a Prospective, Multinational Nonrandomized Trial for Children and Adults With Intracranial Germinoma, Comparing Craniospinal Irradiation Alone With Chemotherapy Followed by Focal Primary Site Irradiation for Patients With Localized Disease. Neuro Oncol (2013) 15(6):788–96. doi: 10.1093/neuonc/not019 PMC3661100 23460321

[24] Foote M Millar BA Sahgal A Ménard C Payne D Mason W. Clinical Outcomes of Adult Patients With Primary Intracranial Germinomas Treated With Low-Dose Craniospinal Radiotherapy and Local Boost. J Neurooncol (2010) 100(3):459–63. doi: 10.1007/s11060-010-0206-9 20455001

